# Agreement and Reliability Analysis of Machine Learning Scaling and Wireless Monitoring in the Assessment of Acute Proximal Weakness by Experts and Non-Experts: A Feasibility Study

**DOI:** 10.3390/jpm12010020

**Published:** 2022-01-01

**Authors:** Eunjeong Park, Kijeong Lee, Taehwa Han, Hyo Suk Nam

**Affiliations:** 1 Integrative Research Center for Cerebrovascular and Cardiovascular Diseases, Yonsei University College of Medicine, Seoul 03722, Korea; eunjeong.ej@gmail.com; 2 Department of Neurology, National Health Insurance Service, Ilsan Hospital, Goyang 10444, Korea; junon8263@gmail.com; 3 Health-IT Center, Yonsei University College of Medicine, Seoul 03722, Korea; taehwa.han@gmail.com; 4 Department of Neurology, Yonsei University College of Medicine, Seoul 03722, Korea

**Keywords:** decision-support system, machine learning, artificial intelligence, sensors, inter-rater reliability, agreement analysis, stroke

## Abstract

Assessing the symptoms of proximal weakness caused by neurological deficits requires the knowledge and experience of neurologists. Recent advances in machine learning and the Internet of Things have resulted in the development of automated systems that emulate physicians’ assessments. The application of those systems requires not only accuracy in the classification but also reliability regardless of users’ proficiency in the real environment for the clinical point-of-care and the personalized health management. This study provides an agreement and reliability analysis of using a machine learning-based scaling of Medical Research Council (MRC) proximal scores to evaluate proximal weakness by experts and non-experts. The system trains an ensemble learning model using the signals from sensors attached to the limbs of patients in a neurological intensive care unit. For the agreement analysis, we investigated the percent agreement of MRC proximal scores and Bland-Altman plots of kinematic features between the expert- and non-expert scaling. We also analyzed the intra-class correlation coefficients (ICCs) of kinematic features and Krippendorff’s alpha of the observers’ scaling for the reliability analysis. The mean percent agreement between the expert- and the non-expert scaling was 0.542 for manual scaling and 0.708 for autonomous scaling. The ICCs of kinematic features measured using sensors ranged from 0.742 to 0.850, whereas the Krippendorff’s alpha of manual scaling for the three observers was 0.275. The autonomous assessment system can be utilized by the caregivers, paramedics, or other observers during an emergency to evaluate acute stroke patients.

## 1. Introduction

The influence of machine learning in medicine has rapidly increased in decision support for detecting symptoms [1]. Most medical artificial intelligence (AI) studies have shown that the promise of medical AI resides in the data used for informing the care of each patient, and the experience and knowledge of experts used for decision making [2]. Even specialists’ decisions can be affected by other factors, including cognitive biases, overconfidence, excessive workloads, and personality traits. Such differences or biases in decision making among specialists and non-specialists can pose a grave problem in care plans, which affects the outcome of treatment, including the mortality of patients [3,4]. To solve this problem, many intelligence solutions have been adopted to reduce medical errors and to meet the efficiency requirements [5,6].

However, the proliferation of medical AI solutions has also raised the debate concerning whether they are reliable in a real environment. As argued by Patrick et al., prematurely released AI solutions can result in increased risks and workloads for clinicians [7]. AI-driven decisions are delicate to maintain the reliability and agreement between healthcare professionals and patients [6]. Specifically, the conversion from patients’ qualitative conditions to quantitative grades in severity scales is confusing and sensitive to determine. As a result, it is challenging to maintain reliability and consistency between graders.

The degree of difficulty in measuring and collecting data determines the availability and the capacity of data for training machine learning models. In addition, ordinal classification suffers from the disparity of data between classes as addressed in [8,9]. To solve this problem, researchers have proposed and achieved prominent solutions to develop high-performance AI systems with the small data set; data augmentation [10,11], transfer learning [12,13], construction of synthetic data [14,15,16] and ensemble learning [17,18,19] are the representative approaches that have resulted in outstanding solutions.

In this study, we investigated the agreement and reliability of automated grading system for non-experts who need to determine the severity of symptoms among patients with acute stroke. The autonomous grading system determines the Medical Research Council (MRC) scale, which is widely accepted and frequently used in a clinical environment to assess neurological conditions and muscle strength (Table 1) [20,21].

The protocol of this study asked acute stroke patients in the neurological intensive care unit to move and hold their limbs for assessing muscle strength, therefore the degree of difficulty in measuring and collecting data is relatively higher than those of studies for patients in rehabilitation or healthy subjects. Data of rare events or important objects are scarce in many studies, therefore, they need pre-processing to lessen the effect of the data capacity in model construction. This skewed distribution usually suffers from a lower performance than the dichotomous classification [22], and it invokes the problem of imbalanced data between classes [23,24]. Most methods are based on the model adjustment and data balancing approaches that have been popularly utilized in applications [25,26]. According to the review research [24], the hybridization of ensemble methods using sampling and cost-sensitive approaches has proven to be an effective solution to the classification with skewed data distribution. Tanha et al. [27] reviewed methods for multi-class imbalanced data classification as data-level methods, algorithm-level methods, and hybrid methods. For the agreement and reliability analysis, we compared the manual and machine learning scaling by investigating the percent agreement, Bland-Altman plot with level of agreement (LoA) and Krippendorff’s alpha for multiclass ordinal classification of MRC grading.

## 2. Materials and Methods

### 2.1. Participants and Study Protocol

**Figure 1:**
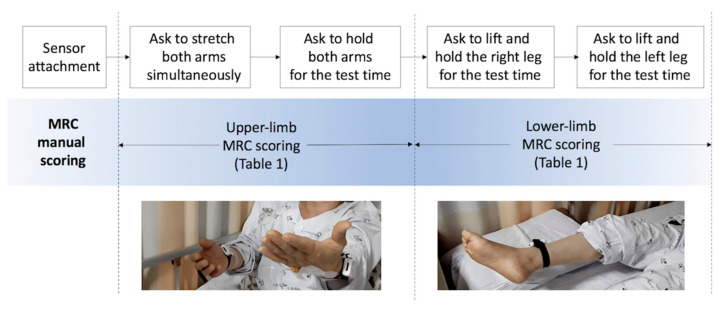
Protocol of proximal-weakness assessment. MRC, Medical Research Council.

We assessed proximal weakness among patients with acute stroke in a neurological intensive care unit on the day they were conscious enough to cooperate. Patients completed drift test trials that measure unintentional drift and oscillation of limbs caused by neurological deficits as shown in Figure 1. The graders asked patients to move and hold their limbs for the test time and scored MRC scales for each limb while inertial sensors objectively measure the movement.

To estimate the symptoms of patients in critical condition, we assessed muscle strength according to the criteria of Table 1 shortly after their admission to a neurological intensive care unit.

We collected 144 sets of inertial sensing data and their MRC gradings and prepared 600 synthetic data sets for training machine learning models. The participants’ ages ranged from 38 to 86 years, with a mean of 65.4 (±16.02) years. The test protocol of manual MRC scoring and objective measurement by sensors attached to the arms and legs of patients are depicted in Figure 1.

For the reliability analysis tests, a neurologist who was familiar with patient assessment observed and coded the MRC scales as the gold standard (GS) labels. The observation window was 20 s to monitor the unintended movement of the limbs. Thereafter, two medical students performed additional tests (TS1 and TS2) for each patient as the assessment by non-experts. The decision-making criteria of the MRC scales were provided to the non-experts as Table 1 in advance of the identical test. The detailed protocol of the drift test for stroke patients is described in the works of [20,28].

The collected data were processed to extract kinematic features representing unintended drift and oscillation caused by neurological deficits. The feature set included the mean drift (MeanDrift), maximum drift (MaxDrift), and accumulated oscillation (SumOsc) during the observation window. The demographic features included gender and age. Three graders’ tests were performed within 24 h. This study was approved by the Severance Hospital Institutional Review Board, and informed consent was obtained from all participants.

### 2.2. Automated Proximal Weakness Scaling

**Figure 2:**
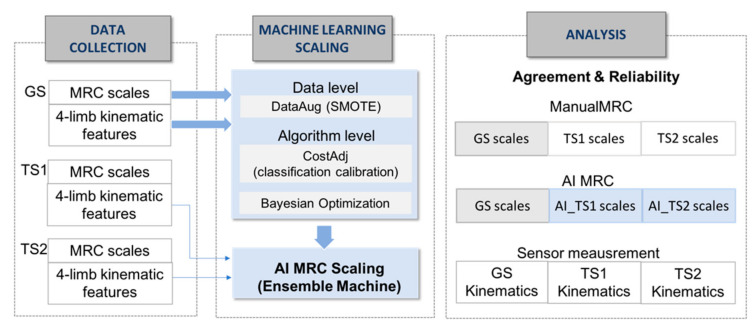
Process of data collection, machine learning-based scaling system and analysis. GS, gold standard; TS1, tester1, TS2, tester2, SMOTE, synthetic minority over-sampling technique.

The data collection in Figure 2 shows the extraction of kinematic features from three graders’ test trials for the machine learning MRC scaling. MRC scaling requires an ordinal classification with skewed distribution in multiple classes. 

To enhance the performance of MRC scaling with insufficient ordinal-imbalanced data, we adopted a hybrid approach of data-level and algorithm-level methods, as shown in the machine learning scaling part of Figure 2; We used synthetic minority over-sampling technique as the data-level method for constructing 600 data sets with class balancing for training the model, adjusting the mislabeling cost for reflecting distances between predicted and actual classes [27]. The data-level method addresses the disparity in data and adds new minority class instances to a training dataset by finding the k-nearest neighbors of a minority class instances and extrapolating between the original instances and its neighbors to create new instances in each iteration. For the training data set *T* = {*T*_1_, *…*, *T_M_*} with a skewed distribution in *M* classes, the algorithm produced a training instance set *SB* with a boosting factor *SBF_i_* for the balanced *N* training instances as follows:

*SB* = {*SB*1, …, *SBM*}

*SBF_i_* = (*N*/*M* − *n*(*T_i_*))/*n*(*T_i_*), (1)

and *n*(*SB_i_*) = *n*(*T_i_*)· *SBF_i,_*

where i= {1, …, M} and *n(T_i_)* represents the number of instances in the *i*th class. We performed the construction of synthetic data set based on the original data *T*, and we trained the ensemble machine using the shuffled data of all the original and synthetic data of *SBT* ($SBT_{i}=SB_{i}\cup T_{i}$).

Given *SBT* with *N* instances with ordered classes, the training algorithm should not only maximize the classification accuracy but also minimize the distances between the actual and predicted classes [22,23]. For this problem, machine learning MRC scaling should adjust the cost matrix, which is expressed in terms of average misclassification costs for the multiple classes. Cost adjustment should express relative and unequal distances between classes to give more penalties for the misclassification that is far from the actual classes. The cost matrix *C*_M×M_ is composed of *C_ij_*, which denotes the penalty that misclassifies a class *j* instance into class *i*. In the linear-weight cost matrix, the cost weights between classes are adjusted as follows:

(2) $$c_{i,j}=\;\left|j-i\right|\;,$$

for *i* = 1, …, *M*, and *j* = 1, …, *M*.

If the classification was not sufficiently improved with data-level methods, the misclassification weight of $C_{M\times M}$ should be corresponding to an imbalance factor as follows [24]:

(3) $$c_{i,j}=\frac{\sum_{i\neq j}^{M}n\left(SBT_{i}\right)}{n\left(SBT_{i}\right)}\left|j-i\right|\;,$$

for *i* = 1, …, *M*, and *j* = 1, …, *M*.

In this study, we adopted an ensemble machine learning and cost adjustment with linear weights for the MRC scales.

After sampling and formulating the cost matrix, we applied the Bayesian optimization algorithm selecting models in ensembles, which attempts to minimize a scalar objective function while selecting and tuning machine learning models [25]. The Bayesian update modifies the Gaussian process model at each new evaluation of the objective function *f(x)*, and the acquisition function *a(x)* based on the Gaussian process model of *f(x)* is maximized to determine the next point *x* for evaluation. In this study, we updated *f(x)* for 50 iterations to select a model among candidate ensemble machines of Bagging, Adaboost, and RUSBoost.

### 2.3. Agreement and Reliability Analysis

Inter-rater agreement and reliability are fundamental to the evaluation of new approaches or methods in various fields [26]. In this study, we calculated the agreement and reliability indices of the kinematic features from measurement and MRC scaling, respectively, as shown in the analysis part of Figure 2. To demonstrate the objective measurement using sensors, we investigated the intraclass correlation coefficients (ICCs) and the Bland-Altman plots with mean-difference and LoA, which are used to compare two measurements of the same variable [27]. Bland-Altman analyses were performed to assess the absolute degree of differences between kinematic features across the entire range of features measured in GS, TS1, and TS2. In the calculation of the ICCs, the average measures and a two-way random model were adopted for each feature [29,30].

To demonstrate the agreement between the expert’s MRC scaling (GS) and machine learning MRC scaling of the non-experts (AI_TS1 and AI_TS2), we investigated the percent agreement between machine learning and GS scaling (GS:AI_TS1 and GS:AI_TS2) [31]. The reliability of machine learning scaling of all the tests was demonstrated using Krippendorff’s alpha [32]. Krippendorff’s alpha is a chance-adjusted index that estimates the level of agreement between graders that can be expected to have occurred by chance [33]. It is known to be superior to other reliability indices because it is applicable to an unlimited number of observers and categories for binary, nominal, and ordinal assessments, and it is robust to missing data [34,35]. We calculated Krippendorff’s alphas to evaluate the reliability of three observers of manual scaling (GS, TS1, and TS2) and machine learning scaling (GS, AI_TS1, and AI_TS2).

Machine learning, including data preparation, tests, and evaluation were performed using Matlab2020a (Mathworks, Natick, MA, USA) [36]. The agreement and reliability were analyzed using the AnnotationTask agreement module in the nltk 3.5 library [37,38].

## 3. Results

### 3.1. Sensor Measurement and Features

**Figure 3:**
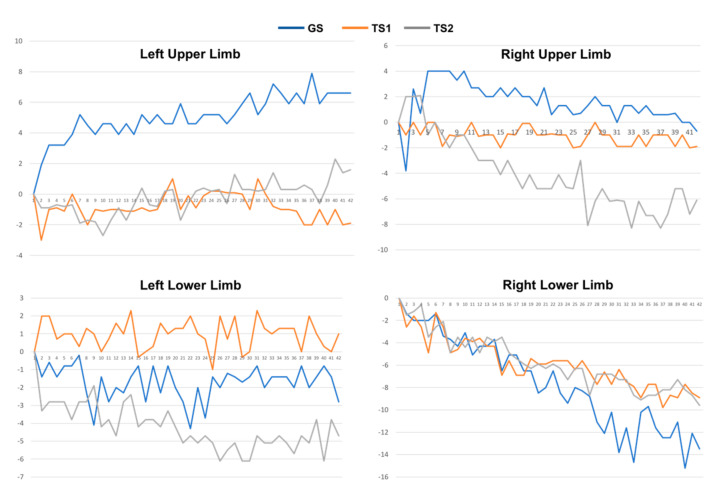
Drift trajectories of 4-limb movement of a patient measured in GS, TS1, and TS2.

**Figure 4:**
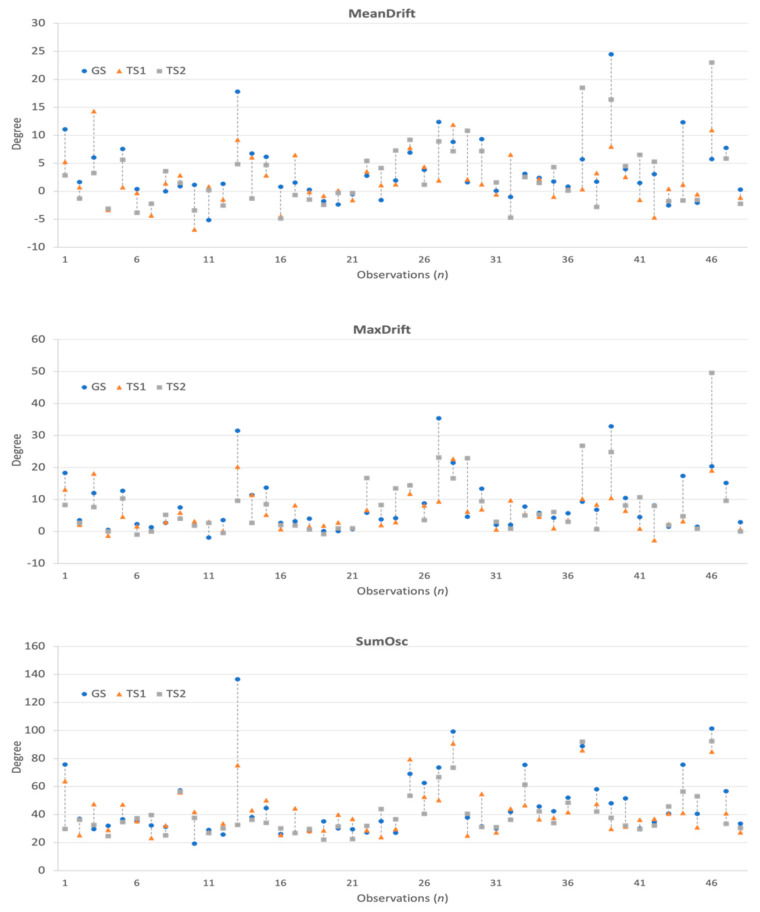
Kinematic features and differences observed in GS, TS1, and TS2.

The collected 3-axis accelerometer signals were converted to the degree of unintentional drift, as shown in Figure 3. Next, for each patient, we collected the drift trajectories measured in GS, TS1, and TS2. The feature extraction process calculates MeanDrift, MaxDrift, and SumOsc from the drift trajectories and the feature values from 144 observations are shown in Figure 4.

**Figure 5:**
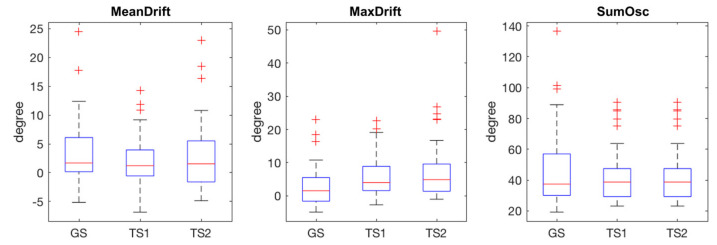
Statistical plots of measured features in GS, TS1, and TS2, red crosses are outliers.

The statistical plots of Figure 5 show the characteristics of the extracted kinematic features. MeanDrift was 3.40 ± 5.57 (GS), 2.03 ± 4.34 (TS1), and 2.82 ± 5.90 (TS2). MaxDrift was 8.39 ± 8.64 (GS), 5.78 ± 5.76 (TS1), and 7.61 ± 9.38 (TS2). SumOsc was 46.82 ± 23.93 (GS), 42.42 ± 17.02 (TS1), and 40.09 ± 15.67 (TS2).

**Figure 6:**
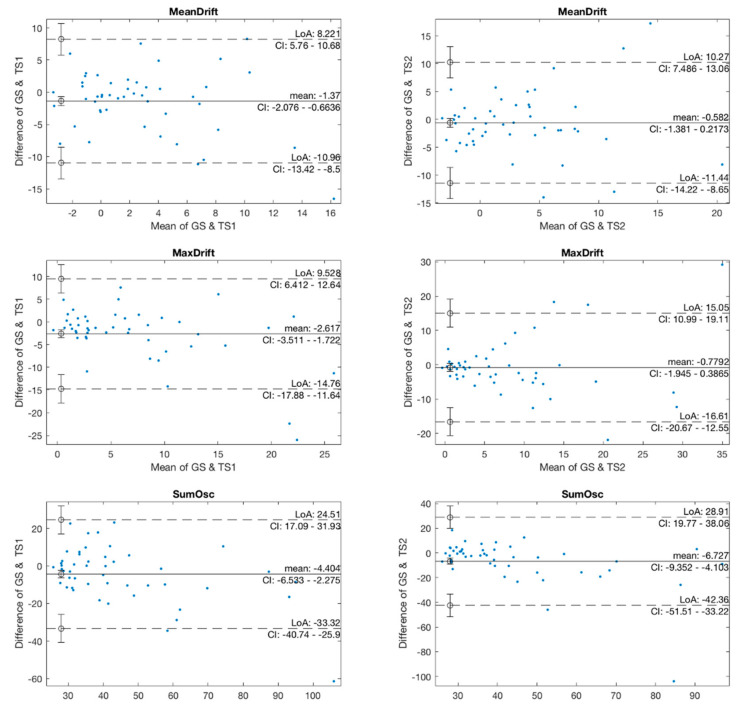
Bland-Altman plots of kinematic features.

In the agreement analysis, we investigated the agreement in kinematic features between two pairs of GS and TS1, and GS and TS2 using Bland-Altman plots, as shown in Figure 6. The mean values in the GS and TS1 plots of MeanDrift, MaxDrift and SumOsc were −1.37, −2.617, and −4.404, respectively, which demonstrated a level of retention showing that the measured values of TS1 were slightly larger than those of GS. The LoA of confidence interval (CI) at 95% was (−10.96, 8.221) for MeanDrift, (−14.76, 9.528) for MaxDrift, and (−33.32, 24.5) for SumOsc, respectively. In the Bland-Altman plot of GS and TS2, the mean values were −0.582 (MeanDrift), −0.7792 (MaxDrift), and −3.825 (SumOsc), and the LoA were (−17; 15.44) (MeanDrift), (−13.28; 11.33) (MaxDrift), and (−17.32; 14,33) (SumOsc), respectively.

The reliability of the kinematic features of the three testers was demonstrated using ICCs as shown in Table 2. The ICCs were 0.742 (MeanDrift), 0.798 (MaxDrift), and 0.850 (SumOsc), which are interpreted as being in the good reliability category (ICC, 0.75–0.90) [39].

### 3.2. Manual and Machine Learning Scaling

**Figure 7:**
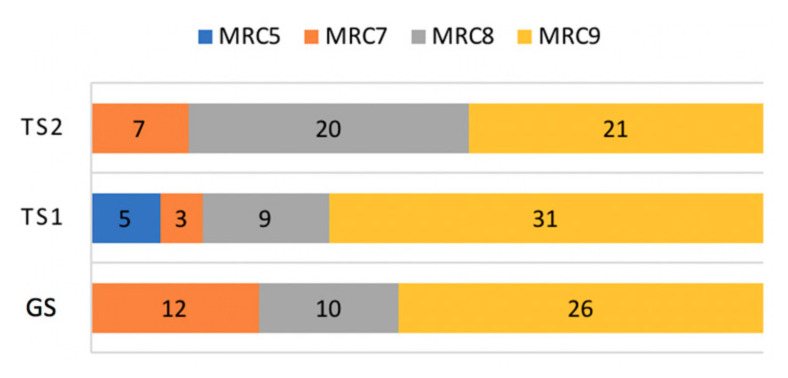
Composition of MRC scales.

The distribution of MRC scales was skewed toward the highest MRC value, with 78 out of 144 observations assessed to MRC 9 (54.2%), as shown in Figure 7. MRC 8, 7, and 5 made up 27.1% (39/144), 15.2% (22/144), and 3.5% (5/144), respectively.

**Figure 8:**
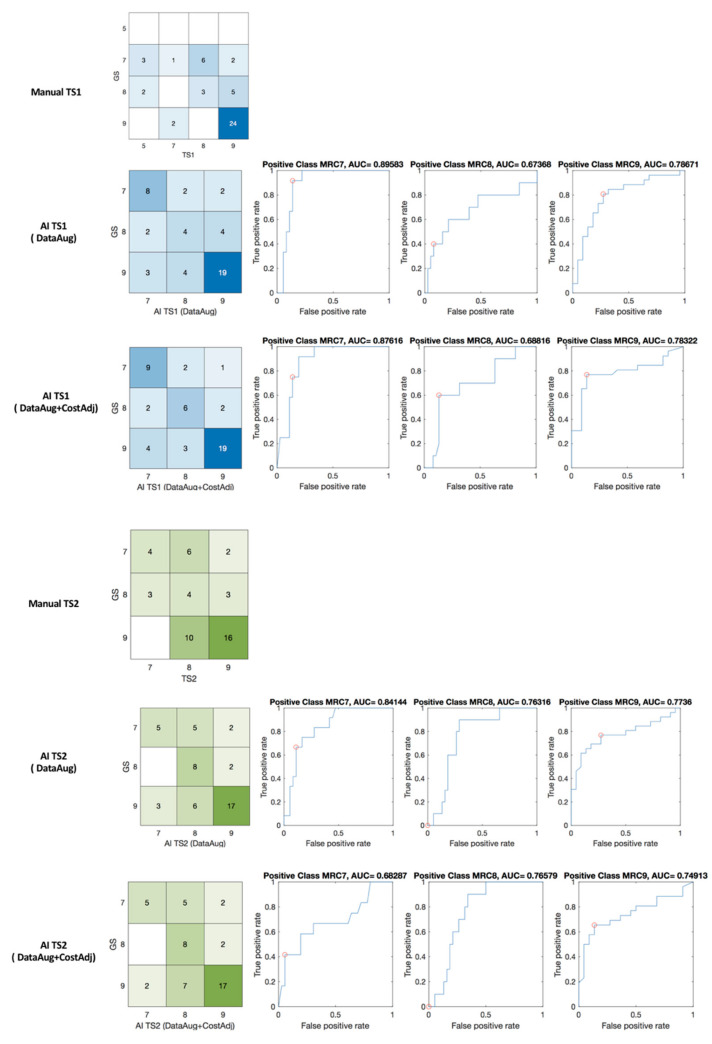
Confusion matrices, ROC of manual (GS: TS1 and GS: TS2) and machine learning (GS: AI_TS1 and GS: AI_TS2) scaling. red circle—optimal operating point in the ROC curve.

The results of MRC scoring by machine learning are shown in Figure 8 with confusion matrices and receiver operating characteristic (ROC) curve and the area under the curve (AUC). The confusion matrix of expert’s scoring and manual scoring of TS1 (GS:TS1) and machine learning grading (GS:AI_TS1) are shown in blue matrices in Figure 8. The percent agreement of between GS and TS1, and GS and AI_TS1 was 0.583 and 0.708, respectively. In TS2, the identical patient group was assessed with a percent agreement of 0.5 for GS and TS2, and machine learning scaling enhanced the index to 0.708 for GS and AI_TS2 as shown in green matrices in Figure 8.

In this study, we more focus on the agreement and reliability of proposed solution than other performance metrics. We analyzed Krippendorff’s alpha of manual and machine learning MRC scaling of the three testers, as shown in Table 3.

Krippendorff’s alpha values are interpreted as fair in manual scaling with 0.291 for GS-TS1, 0.206 for GS-TS2, 0.275 for GS-TS1-TS2. The enhanced reliability with machine learning scaling was interpreted as moderate when it was hybridized through data augmentation (K-alpha 0.422 and 0.407 for GS-AI_TS1 and GS_AI_TS2, respectively). The reliability of machine learning scaling with data augmentation and cost adjustment increased more to 0.537 for GS-AI_TS1, 0.405 for GS-AI_TS2, and 0.455 for GS-AI_TS1-AI_TS2. Consequently, the machine learning scaling with data augmentation and cost adjustment increased the reliability index of the three testers’ MRC scaling from 0.275 to 0.445.

## 4. Discussion

### 4.1. Agreement and Reliability of AI Model in Clinical Decision Making

Many promising results have accelerated the application of AI in medicine, which assists in making clinical decisions and saving personnel and time during the provision of care. Many studies argue that the first potential roles of medical AI involve triage situations or screening tools [1,40,41]. Specifically, AI prediction with patient monitoring is crucial in real-world environments, such as intensive care units, emergency rooms, and cardiac wards, where timeliness in clinical decision-making can be measured in seconds [6]. In experiments carried out in static environments or in simulations, many medical AI solutions demonstrate impressive performance. Therefore, they still need to be translated to work in realistic clinical settings [42] because the successful implementation of medical AI is premised on its reliable performance in real-world applications. Otherwise, the reliability problem results in the resistance to the application of medical AI, with the concerns being safety and performance [43,44]. To ensure the robust evaluation of performance in a real environment, agreement and reliability are significantly essential factors affecting the adoption of AI-based approaches in medicine. However, many researchers often focused solely on the accuracy of prediction in the use of AI, and they use the terms ‘reliability’ and ‘agreement’ interchangeably despite their technical distinction [26,45]. The selection of a proper evaluation method for reliability is significant in adopting a new approach [7,26,33]. As argued by Nili et al., the evaluation methods for reliability should be determined by considering the type of data, including nominal, ordinal, interval, and ratio data; Missing data of observation, the number of observers, and minimizing the effect of chance in agreement should also be considered [46].

In this pilot study, the Bland-Altman plots and ICCs were used to indicate the agreement and reliability of measured movement through sensors. As shown in the results, the ICCs of the kinematic features between GS, TS1 and TS2 were high (0.742–0.850). Nevertheless, the agreement of manual scaling to the GS was 0.5 for TS1 and 0.508 for TS2. This is caused by the difference in grading qualitative conditions into quantitative scales with cognitive bias in experience. The assessment of facial palsy is also one of the medical decisions that require the recognition of subtle differences among symptoms, as shown in the study on reliability in MRC scaling [21].

For the evaluation of reliability in multiple tests, a Krippendorff’s alpha was used to indicate the reliability between the tests of graders. A negative alpha indicates an inverse agreement less than that expected by chance. Our findings show that the reliability of manual scaling was in the range of fair reliability (0.21–0.40), and the index of machine learning scaling (0.537) increased to moderate inter-rate reliability (0.41–0.60) using the hybrid approach of data augmentation and cost adjustment [32]. Although the reliability indices in this study are still lower than those in dichotomous classification, the enhancement was demonstrated through machine learning and data preprocessing techniques with respect to the equivocal triage categories; The percent agreement was also enhanced with an average improvement of 30.63%.

This study is to investigate the feasibility of autonomous grading as a consistent and reliable tool to estimate the assessment performed by an expert. To utilize the system as a standard tool, we need to extend the clinical trials to include data from multiple institutes in future works.

### 4.2. Developing AI for Personalized Medicine with Disparity and Insufficiency of Data

The quality of AI-based decisions is determined by the amount of high-quality training data. However, the availability of data that is representative of the target patient population depends on the environment, urgency, patient conditions, and the availability of facilities for compiling data [6]. As addressed in many studies, AI research in the real environment has the long-tail problem in which data of rare and important events or objects are scarce [8,9,47]. The same problem appears with the availability of medical data, where data extracted from hospital information systems or health data from daily life monitoring are relatively easy to collect and provide sufficient data, however, the systems for monitoring and assessing acute symptoms, especially systems that require patients to actively participate in data collection, suffer from scarce data. The sensor data of limb movement for patients with acute stroke in the intensive care unit, which we measured in this study, were also hard to collect because stroke patients are asked to be attached sensors on the body and to actively stretch and hold limbs to follow protocols after thrombolysis. Consequently, collecting the movement data of emergency patients requires careful interruptions in the streamline of care, and is harder than fetching medical records from hospital information systems or public open data.

To overcome the limitation of small data set, various techniques have been applied and achieved promising results. Data augmentation and transfer learning are representative solutions for deep neural networks [10,11,12,13], and synthetic data and ensemble machine learnings also showed prominent performance in the studies with small data sets for rare diseases and acute symptoms [14,15,16,17,18,19].

In the systems for the treatment of acute symptoms, not only data collection but also the application of autonomous systems results in the inevitable intervention into the current streamline of treatment. Therefore, interventions involving medical AI solutions need to demonstrate their impact on health outcomes by undergoing rigorous and prospective evaluation, as addressed by He and Garcia [39]. The medical AI for acute diseases needs to be deliberately designed for application in real-world environments. Although AI for diagnosis or automatic detection in medical images have achieved promising results [48,49,50,51], the application of AI in the treatment of acute diseases should meet the requirements not only to support experts, but to also ensure the timeliness of decisions that face obstacle involving the insertion of new systems in the pipeline of treatment. However, the need for a reliable and consistent assessment during emergencies to save time have been addressed, and the corresponding applications and protocols continuously have been developed [52,53].

## 5. Conclusions

We demonstrated that machine-learning scaling achieved substantial improvement in inter-rater reliability for assessing proximal weakness in clinical scores. The improved agreement in patient assessment between observers can reduce medical errors during decision-making, especially during communication in the streamline of treatment in which experts and non-experts with various roles of care are involved. In our analysis, non-expert assessment with objective measurement using sensors and machine-learning-based scoring improved agreement and reliability. This can improve the grounded application of reliable AI in the streamlining of care.

## Figures and Tables

**Table 1:** Modified MRC scales for assessing proximal weakness. MRC, Medical Research Council.

MRC Scale (6-Point Scale)	Response
9 (V)	Normal power
8 (IV+)	Muscle holds the joint against a combination of gravity and moderate resistance, but muscle holds the joint against moderate to maximal resistance
7 (IV)	Muscle holds the joint against a combination of gravity and moderate resistance
6 (III+)	Muscle holds the joint against a combination of gravity and moderate resistance, but muscle holds the joint only against minimal resistance
5 (III)	Muscle moves the joint fully against gravity and is capable of transient resistance, but collapses abruptly
4 (II+)	Muscle cannot hold the joint against resistance, but moves the joint fully against gravity
3 (II)	Muscle moves the joint against gravity, but not through full mechanical range of motion
2 (I+)	Muscle moves the joint when gravity is eliminated
1 (I)	A flicker of movement is observed or felt in the muscle

**Table 2:** Intra-class correlation coefficients (ICCs) of kinematic features (2-way random).

Feature	ICC (2, k)	*p*	CI (95%)
MeanDrift	0.742	<0.001	(0.59–0.85)
MaxDrift	0.798	<0.001	(0.68–0.88)
SumOsc	0.850	<0.001	(0.76–0.91)

**Table 3:** The Krippendorff’s alpha (K-alpha) and Fleiss kappa of manual and machine learning scaling.

Methods	Metrics	GS-TS1	GS-TS2	GS-TS1-TS2
Manual	K-alpha	0.291	0.206	0.275
	Fleiss Kappa	0.300	0.218	0.285
Machine Learning (DataAug)	K-alpha	0.422	0.407	0.381
	Fleiss Kappa	0.416	0.413	0.383
Machine Learning (DataAug + CostAdj)	K-alpha	0.537	0.405	0.445
	Fleiss Kappa	0.534	0.414	0.448
